# The Differential Diagnosis of Multiple Glandular Swellings—I

**Published:** 1908-12-19

**Authors:** 


### Medicine.

### THE DIFFERENTIAL DIAGNOSIS OF MULTIPLE GLANDULAR SWELLINGS—I

It not infrequently happens that one of the main features of a case is enlargement of the cervical, axillary, or inguinal glands, and the immediate question for decision will be, What is the nature of the disease and the cause of these multiple glandular swellings?

The conditions that will occur to one as possibilities are: (1) Tubercle, (2) Hodgkin's disease, (3) lymphadenoma, (4) lymphosarcoma, (5) lymphatic leuchæmia, (6) secondary squamous-celled or other carcinoma. Simple inflammatory glands, secondary syphilis, and German measles will, as a rule, be obvious enough not to need discussion.

The points upon which the differential diagnosis will be based are: (*a*) the blood count; (*b*) the history of how the glands developed; (*c*) the condition of the glands themselves; (*d*) the age of the patient; (*e*) the condition of the spleen; (*f*) the presence or absence of signs of enlarged mediastinal glands; (*g*) Calmette's and von Pirquet's tuberculo-reactions; (*h*) excision and microscopical examination of a gland; (*i*) the course of the disease.

(*a*) *The Blood Count.*—This is placed first in the above list because, whether it be made at the beginning or only at a later stage in the diagnosis, it is in all cases essential. No case of general enlargement of the lymphatic glands has been thoroughly investigated unless the blood has been examined, although there is only one of the affections named above in which the blood count is pathognomonic—namely, lymphatic leuchæmia. If it is found that there are 100,000 leucocytes or more per cubic millimetre of blood, and that in the differential leucocyte count there are 90 per cent. of lymphocytes, then the disease is lymphatic leuchæmia, no matter what the red corpuscles show, and no matter what other features the case presents. If there is no such lymphocytosis, then lymphatic leuchæmia is excluded, and it is for exclusion of this disease that the blood count is essential in all cases. In all the other diseases that cause generalised enlargement of the lymphatic glands the blood characters are negative. They may be perfectly normal. More often the blood presents moderate anæmia of the chlorotic type without much change in the leucocytes. Sometimes, with or without chlorotic anæmia, there may be a relative increase in the small lymphocytes in some cases of Hodgkin's disease and in some of tuberculous adenitis. The following, for example, is a count which might suggest Hodgkin's disease—in which it actually occurred—though not absolutely pathognomonic:

| | |
|---|---|
| Red corpuscles ... ... | 2,800,000 per cub. mm. |
| Hæmoglobin ... ... ... | 49 per cent. |
| Colour index ... ... ... | 0.877 |
| Leucocytes ... ... ... | 5,800 per cub. mm. |

Differential leucocyte count:

| | |
|---|---|
| Small lymphocytes ... ... ... | 36 per cent. |
| Large lymphocytes ... ... ... | 8 ,, |
| Polymorphonuclear cells ... | 53 ,, |
| Coarsely granular eosinophile cells ... | 1.0 ,, |
| Basophile cells ... ... ... ... | 0.5 ,, |
| Myelocytes ... ... ... ... ... | 1.5 ,, |

(*b*) *The History.*—This may clearly be of importance in many ways. If the glands in the neck came first there are a greater number of possibilities than if those in the groin were the first. If the glands became enlarged very quickly, the mischief is more likely to be inflammatory, leuchæmic or malignant, whereas if the enlargement has been slowly coming on for years, it is clear that leuchæmia and secondary carcinoma are excluded, whilst tubercle and Hodgkin's disease are both still possible, the former being the more likely of the two. The patient may be quite sure that the lumps have "come and gone" several times; this would be unlikely except in the case of inflammatory or tuberculous glands.

(*c*) *The Condition of the Glands Themselves.*—The first point to pay attention to in regard to the glands themselves is whether the axillary and inguinal glands are quite materially enlarged, as well as those in the neck, or whether, the cervical glands having been found big, examination has not revealed palpable axillary and inguinal glands which may be slightly larger than they should be, and yet are not so decidedly big that they would have attracted notice if the cervical glands had not been observed first. This is a very real point in actual practice. It often happens that one wonders whether or not the palpable axillary or inguinal glands are really enlarged when there is no doubt about the enlargement of those in the neck.

If there is no doubt whatever about all three groups of glands being enlarged, then there are only three likely possibilities—namely, Hodgkin's disease, lymphadenoma, and lymphosarcoma. Secondary carcinomatous glands might occur in axilla and neck, in a case of cancer of the breast for example, but not in the groin as well. Tuberculous glands are very seldom found in neck, armpit, and groin in the same patient, though many a case in which there are tuberculous cervical glands is so thin that one may mistake merely palpable axillary and inguinal glands for enlarged ones.

The distinction between Hodgkin's disease, lymphadenoma, and lymphosarcoma is very largely an arbitrary matter. There are some authorities who lay down points of absolute distinction between the three conditions, but if a whole series of cases be examined every stage of transition between one type and another will be found, so that it is more probable that they are really all the same disease under different aspects. The main points of distinction usually made between them depend upon two factors—namely, (1) whether or not the spleen is enlarged, (2) whether a longer time or a shorter elapses between the onset of the first symptom and the death of the patient. Thus the three types would be:

*Lymphadenoma.*—Generalised enlargement of the lymphatic glands, one group usually being much more affected than the others; no enlargement of the spleen; survival of the patient for several years.

*Lymphosarcoma*, or malignant lymphadenoma; precisely similar to lymphadenoma, except that only weeks, or at most months, elapse between the first symptom and the patient's death.

*Hodgkin's Disease.*—Generalised enlargement of the lymphatic glands, one group usually being much more affected than the others; enlargement of the spleen, in which the Malpighian corpuscles are seen post-mortem to be so greatly hypertrophied that the cut section of the organ has been compared to hard-bake—death either in a few weeks, or in a few months, or in a few years, according as the disease is acute, subacute, or chronic— the latter being the commonest of the three.

